# *ERCC3* Gene Associated with Breast Cancer: A Genetic and Bioinformatic Study

**DOI:** 10.1155/2024/7278636

**Published:** 2024-07-14

**Authors:** Xiangyu Chen, Heng Xiao, Shuangcheng Ning, Bang Liu, Huashan Zhou, Ting Fu

**Affiliations:** ^1^ Department of Pathology Changsha Hospital for Maternal and Child Health Care Hunan Normal University, Changsha, Hunan, China; ^2^ Hunan Provincial Key Laboratory of Regional Hereditary Birth Defects Prevention and Control Changsha Hospital for Maternal and Child Care Hunan Normal University, Changsha, Hunan, China; ^3^ Department of Pathology School of Medicine Hunan Normal University, Changsha, Hunan, China

## Abstract

Female breast cancer is the most common and the fifth deadliest cancer worldwide. It is influenced by a combination of genetic, hormonal, and environmental factors. The excision repair cross-complementation group 3 gene (*ERCC3*) has recently been identified as a breast cancer susceptibility gene in various cohorts of different geographical and ethnic origin. To explore the role of *ERCC3* mutations in breast cancer development and pathological diagnosis, genetic analysis was conducted in 291 patients and 291 controls from mainland China. Bioinformatic analysis and immunohistochemistry (IHC) were performed. A novel *ERCC3* mutation p.Y116X was identified in a breast cancer family, while no frequency bias for the genotype and allele of rs754010782 and rs371627165 was observed (all *P* > 0.05). Bioinformatic analysis revealed that *ERCC3* expression was negatively associated with estrogen receptor (ER), progesterone receptor (PR), nontriple-negative status, and nodal status of breast cancers. *ERCC3* amplifications and deep deletions primarily occurred in breast invasive cancer not otherwise specified (NOS) and metaplastic breast cancer, respectively. The decreased ERCC3 expression in tumor tissues of patient with p.Y116X mutation was found by IHC. The *ERCC*3 mutation p.Y116X may increase breast cancer risk in the Han-Chinese population. *ERCC3* exhibits potential as a biomarker for the pathological diagnosis of breast cancer.

## 1. Introduction

Female breast cancer is the most common and the fifth deadliest cancer, accounting for an estimation of 2.26 million newly diagnosed cases and 6.85 million new cancer-related deaths worldwide yearly [1]. It is a heterogeneous disease and its incidence is under the interactive effect of genetic, hormonal, and environmental factors [2]. Breast cancer diagnosed through histopathological analysis in female under the age of 40 years old are defined as early-onset breast cancer which is strongly linked to aggressive triple-negative or human epidermal growth factor 2 (HER2)-positive tumors [3]. Following a four-generation family with multiple female members affected firstly reported in 1866, a series of epidemiological studies recognized genetic as an important risk factor for breast cancer, which explained approximately 5%∼10% of the patients [4]. In recent decades, the advancement of candidate gene-association studies and genome-wide association studies (GWASs), along with meta-analysis of multiple GWAS, has greatly expedited the identification of susceptibility single nucleotide polymorphisms (SNPs), which have been estimated to contribute to approximately 41% of the heritability of breast cancer [5–7]. In addition, more than half of familial cases are of undefined genetic cause [8]. Exploration of the genome of these cases may benefit the genetic underpinnings of breast cancer and bring advances in genetic counselling, cancer-risk assessment, cancer prevention, and patient management.

In recent years, the excision repair cross-complementation group 3 gene (*ERCC3*) was proposed as a susceptibility gene for breast cancer. Genetic heterogeneity and population-specific frequencies of *ERCC3* mutations were observed in cohorts from diverse geographical and ethnic backgrounds [9–12]. To identify *ERCC3* mutations that correlate with breast cancer in the Han-Chinese population, genetic analysis of the *ERCC3* gene was performed in 291 Han-Chinese breast cancer patients and 291 cancer-free controls by Sanger sequencing, along with a bioinformatic analysis of breast cancer data obtained from various databases.

## 2. Materials and Methods

### 2.1. Subjects and Pathological Diagnosis

We enrolled a total of 291 Han-Chinese female patients newly diagnosed with breast cancer (mean age: 48.430 ± 13.822 years). Among them, a cohort of early-onset breast cancers consisted of 97 unrelated sporadic cases and 26 familial patients without *BRCA* mutations (onset age range: 21∼40 years, at least one close relative diagnosed with breast cancer before the age of 40 or two close relatives diagnosed before the age of 50) [13]. Available relatives of the patients with *ERCC3* germline variants were involved for the mutation analysis of pedigree. All patients enrolled in the study were histopathologically confirmed breast cancer based on the 2019 World Health Organization (WHO) classification of tumors of the breast by three independent pathologists from Changsha Hospital for Maternal and Child Health Care, Hunan, China [14]. To create a highly comparable control group, the inclusion criteria were set as Han-Chinese female aged 20∼75 years who have no medical record of cancer. Cases and controls were matched by age (±2 years) at a ratio of 1 : 1. Finally, 291 Han-Chinese female controls with similar age distribution (mean age: 49.113 ± 13.694 years, *P* = 0.451) were included. This study was approved by the Institutional Review Board of Changsha Hospital for Maternal and Child Health Care, Changsha, Hunan, China (approval number: 2022035).

### 2.2. DNA Extraction and Quantification

To identify the *ERCC3* germline variants in early-onset breast cancers and controls, germline genomic DNA was extracted from peripheral blood leukocytes using QIAamp DNA Blood Mini kit (Qiagen, Germany). For the other breast cancers, *ERCC3* germline/somatic variants were detected by extracting genomic DNA from formalin-fixed and paraffin-embedded (FFPE) tumor tissues using the QIAamp FFPE Tissue Kit (Qiagen, Germany). All tumor tissues were fixed in 10% neutral buffered formalin for less than 8 hours. The FFPE blocks preserved within 48 hours were used for DNA extraction. Ten 10-*μ*m FFPE sections of each tumor tissue were manually microdissected, enriching neoplastic cellularity to at least 70%. Following variants identification, germline genomic DNA was also extracted from relatives of early-onset breast cancers with *ERCC3* germline variants. The purity of DNA was further evaluated by NanoDrop™ 8000 spectrophotometer (Thermo Scientific, USA). The OD260/280 ratios ranging from 1.8 to 2.0 were deemed acceptable. All procedures were processed referring to manufacturer's instructions.

### 2.3. Genetic Analysis

Primers for all exons and exon-intron junctions of the full-length NM_000122.2 mRNA transcript were designed online by using Primer 3 (https://bioinfo.ut.ee/primer3-0.4.0/). All primers were synthesized by Sangon Biotech (Sangon, China) (Table 1). The amplification of the *ERCC3* gene through the polymerase chain reaction (PCR) was conducted using the SimpliAmp PCR Thermal Cycler (Thermo Scientific, USA). The PCR conditions consisted of a 3-minute denaturation phase at 98°C, 35 cycles of 10 seconds at 98°C, 10 seconds at 58°C, 15 seconds at 72°C, and a final 5-minute extension phase at 72°C. Each PCR reaction was conducted with a total volume of 25 *μ*L. The reaction mixture consisted of 1 *μ*L of genomic DNA, 1 *μ*L of forward primer, 1 *μ*L of reverse primer, 0.32 *μ*L of deoxyribonucleoside triphosphates mix, 0.15 *μ*L of Taq DNA polymerase, 2 *μ*L of MgCl_2_, 2.5 *μ*L of 10 × PCR buffer, and 17.03 *μ*L of DNase/RNase-Free distilled water. PCR products were purified using the AxyPrep PCR Clean-up Kit (Axygen, USA) and subsequently subjected to sequencing on an ABI 3500 Genetic Analyzer (Applied Biosystems, USA) employing the BigDye XTerminator Kit.

The coding regions of *ERCC3* in breast cancers were sequenced in order to screen for variants. Variant validation was performed on the controls and patients' relatives by Sanger sequencing on candidate exomes. DNAMAN (version 9) was employed for conducting multiple sequence alignment, while ChromasPro (version 2.1.3) was utilized for sequencing analysis. Variants in this study were designated in accordance with the guidelines of the Human Genome Variation Sequence systematic nomenclature (HGVS, https://www.hgvs.org/). Allelic frequencies of variants were examined by referencing with population databases including the Exome Aggregation Consortium, the 1000 Genomes Project, the Genome Aggregation Database, and the China Metabolic Analytics Project (ChinaMAP).

### 2.4. Statistical Analysis and Bioinformatic Analysis

The genotypic frequency distribution among the control group was calculated in order to test for Hardy–Weinberg equilibrium using Pearson's chi-square tests. Differences in genotypic and allelic frequency of variants between patients and controls were compared using Fisher's exact tests, and a two-sided *P* value less than 0.05 was considered as statistically significant. Sequence variant interpretations were acquired through the utilization of *in silico* predictive algorithms including MutationTaster, Polymorphism Phenotyping v2, protein analysis through evolutionary relationships, and functional analysis through Hidden Markov models. The American College of Medical Genetics and Genomics (ACMGs) standards and guidelines for sequence variants interpretation were used to evaluate the functional significance of variants. The structures of the wild-type and mutant proteins were modeled using SWISS-MODEL (https://swissmodel.expasy.org/interactive) and visualized with PyMol (version 2.6.0a0). Relationship between the *ERCC3* gene expression and cancer clinicopathological features was analyzed using the Breast Cancer Gene-Expression Miner v4.9 (bc-GenExMiner v4.9, https://bcgenex.ico.unicancer.fr/BC-GEM/GEM-Accueil.php?js=1) online tool. The cBio Cancer Genomics Portal (c-BioPortal, https://cbioportal.org) software was employed to explore genomic alterations of the *ERCC3* gene in breast cancer populations.

### 2.5. Immunohistochemistry (IHC)

The 4-*μ*m sections from FFPE tissues of patients with *ERCC3* variants and cancer-adjacent normal breast tissues without *ERCC3* alteration were cut, mounted, and deparaffinized. Heat-induced epitope retrieval was performed on sections using sodium citrate buffer (10 mmol/L, pH 6.0) at 110°C for 20 minutes, followed by a 20-minute cooling-off period. The sections were treated with 3% BSA for 10 min to reduce nonspecific staining. Primary antibodies against *ERCC3* (XPB rabbit polyclonal antibody from Invitrogen, ThermoFisher, USA) was diluted at a ratio of 1 : 100 and incubated overnight at 4°C. For secondary antibody incubation, MaxVision™ HRP-Polymer anti-Rabbit IHC Kit (MXB biotechnologies, China) was used. The sections were incubated with the secondary antibody at room temperature for 15 minutes. To visualize the antibody-antigen interaction, diaminobenzidine chromogenic procedures were performed using the DAB PLUS Kit (MXB biotechnologies, China) for 4 minutes. Hematoxylin staining was then conducted for 10 seconds. Breast cancer tissues without *ERCC3* alteration were selected to serve as positive external control, and negative controls were obtained by replacing primary antibodies with phosphate-buffered saline.

## 3. Results

**Figure 1:**
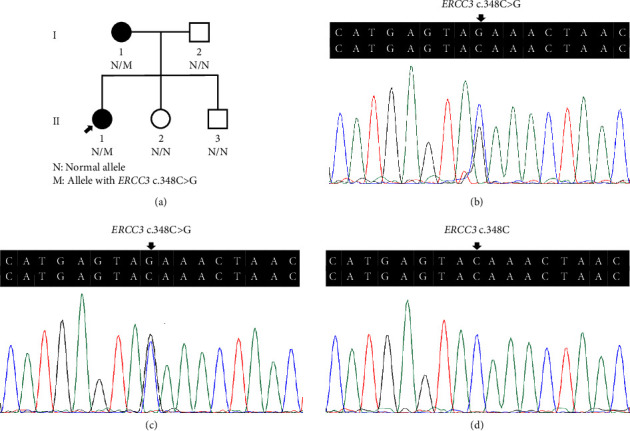
The genetic findings of breast cancer family in the study. (a) Pedigree of the family with hereditary breast cancer. (b) Chromatogram of the germline heterozygous *ERCC3* mutation c.348C > G in the proband (II: 1, P-1). (c) Chromatogram of the mutation in the affected member (I: 1). (d) Chromatogram of the wild-type *ERCC3* gene in the healthy member (II: 2).

**Figure 2:**
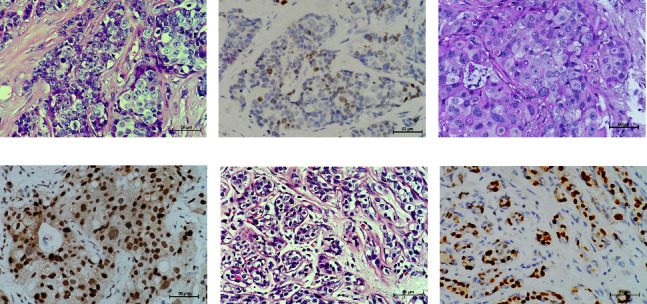
The hematoxylin-eosin staining and *ERCC3* IHC in breast tissues. (a) The specimen of the P-1 was diagnosed to nonspecific invasive carcinoma (grade III) in the right breast. (b) Low expression of *ERCC3* in the tumor tissues of the P-1 by IHC. (c) The hematoxylin-eosin staining in positive external control. (d) *ERCC3* expression in positive external control by IHC. (e) The hematoxylin-eosin staining in normal breast tissues. (f) Wild-type *ERCC3* expression in normal breast tissues by IHC.

Three variants including a novel nonsense variant (c.348C > G, p.Y116X) and two rare missense variants (c.1078C > T, p.R360C, rs754010782; c.1411G > A, p.V471I, rs371627165) were detected in the breast cancers. The frequencies of these variants in the control group were found to adhere to Hardy–Weinberg equilibrium, with all *P* values exceeding 0.05. The allelic frequencies of the variants in public databases, along with their functional predictions, suggest a possible clinical significance (Table 2). The nonsense variant, c.348C > G (p.Y116X), was identified in the proband (P-1) and not contained in population databases. In her family, the mother was diagnosed with nonspecific invasive carcinoma (grade II) in the right breast at the age of 38. This variant cosegregated with breast cancer in the family by germline mutation analysis (Figures 1(a), 1(b), 1(c), and 1(d)). The pathological findings of breast cancer in the proband and her affected mother were displayed (Figure 2(a) and Supplementary Figure 1). The rs754010782 and rs371627165 were identified in sporadic patients (rs754010782 in S-1 and S2; rs371627165 in S-3, S-4, and S-5).

The clinicopathological characteristics of 6 patients were summarized (Table 3 and Supplementary Figures 2 and 3). The distribution of genotypic frequencies and allelic frequencies are presented in Table 4. No statistically significant differences for genotypic and allelic frequencies of rs754010782 (genotype: *P* = 0.499; allele: *P* = 0.500) and rs371627165 (genotype: *OR* = 3.021, 95% *CI* = 0.312∼29.212, and *P* = 0.624; allele: *OR* = 1.503, 95% *CI* = 0.250∼9.026, and *P* = 1.000) were observed between the patients and controls. According to the ACMG standards and guidelines, “likely pathogenic” was tagged to p.Y116X while p.R360C and p.V471I were classified as “uncertain significance.”

**Figure 3:**
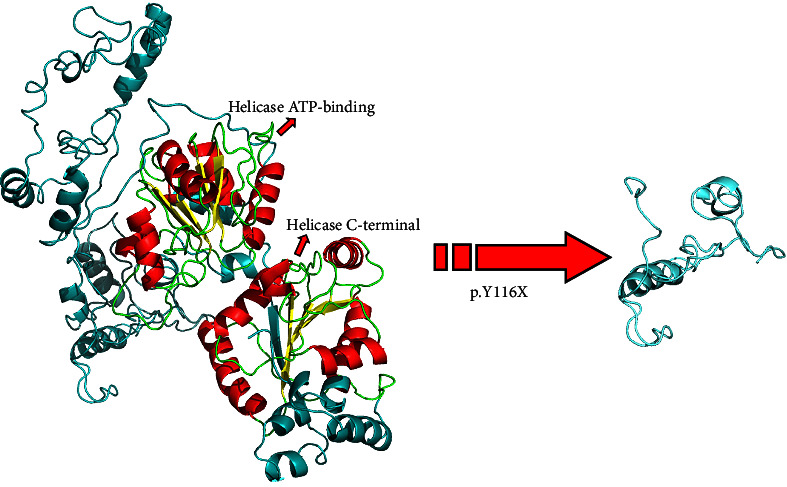
The three-dimensional structure of wild-type and mutant *ERCC3* protein.

**Figure 4:**
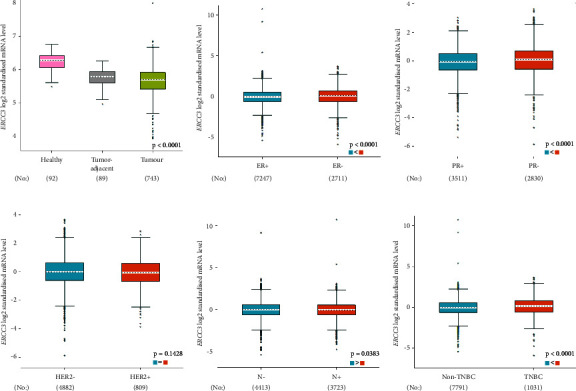
Box plot of the relationship between *ERCC3* expression and different clinicopathological features using bc-GenExMiner v4.9 software. Analysis according to (a) nature of tissues, (b) ER, (c) PR, (d) HER-2, (e) nodal status, and (f) triple-negative status.

**Figure 5:**
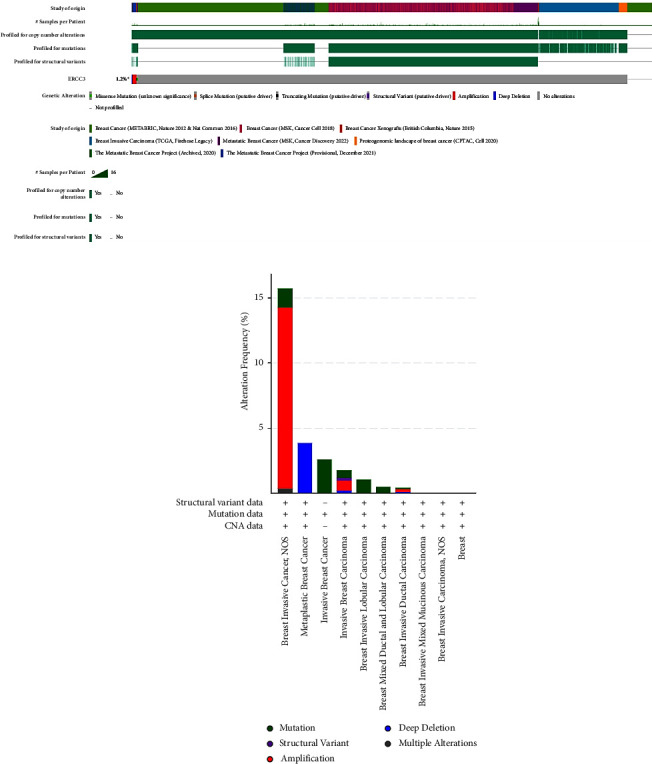
Genetic alterations of the *ERCC3* gene in breast cancer by c-BioPortal software. (a) Summary of *ERCC3* genomic alterations. (b) Frequencies of *ERCC3* alterations in the different clinical subgroups.

The p.Y116X truncated the *ERCC3* protein and caused the loss of helicase ATP-binding domain and helicase C-terminal domain (Figure 3). Analysis of ribonucleic acid sequencing data from the Cancer Genome Atlas and the Genotype-Tissue Expression by bc-GenExMiner v4.9 demonstrated a decrease in *ERCC3* expression in tumor tissues compared to both tumor-adjacent and healthy tissues (Figure 4(a)). DNA microarrays data analysis indicated that the estrogen receptor (ER) and progesterone receptor (PR) status had a negative association with *ERCC3* expression (Figures 4(b) and 4(c)), and the human epidermal growth factor receptor-2 (HER-2) status seemed to be independent of *ERCC3* expression (Figure 4(d)). In addition, decreased *ERCC3* expression was observed in patients with positive nodal status (Figure 4(e)). The nontriple-negative breast cancer group also displayed a lower level of *ERCC3* expression than the triple-negative breast cancer group (Figure 4(f)). A total of 7101 breast cancer patients from 8 studies were involved in analysis by c-BioPortal, and 1.2% had *ERCC3* gene genomic alterations (Figure 5(a)). Amplification of *ERCC3* primarily occurred in breast invasive cancer not otherwise specified (NOS), while most of deep deletions occurred in metaplastic breast cancer (Figure 5(b)).

In the tumor tissues of patient P-1, IHC exhibited a faint nuclear staining in <10% of cells (Figure 2(b)). The normal staining of positive external control confirmed that the IHC results were the standard (Figures 2(c) and 2(d)). The IHC staining of normal breast tissues revealed a moderate to strong staining of wild-type *ERCC3* expression (Figures 2(e) and 2(f)). Diffuse and mild *ERCC3* staining was observed in the tumor tissues of patient S-1, S-2, S-3, and S-5. Diffuse and moderate staining was present in the tumor tissues of patient S-4. IHC analysis indicated an obviously *ERCC3* negative in the tumor tissues of the patient with p.Y116X mutation, while in tissues of patients with variants of uncertain significance, mild or moderate staining nucleus in >80% of cells was found (Supplementary Figures 2 and 3).

## 4. Discussion

The breast cancer susceptibility gene variants are considered the most significant familial risk factor for the occurrence of this disease, particularly in early-onset cases [15]. With the knowledge of today, about a proportion of 5%∼10% breast cancers occur due to genetic risk, and pathogenic variants in breast cancer susceptibility genes are responsible for 25%∼30% hereditary cases [16]. However, over half of hereditary cases without variants in these genes indicate a missing heritability in breast cancer genetics [8]. The *ERCC3* gene encodes an ATP-dependent 3′ to 5′-directed DNA helicase that plays a crucial role in basal RNA transcription and the nucleotide excision repair (NER) pathway as the p89 subunit of the human transcription factor IIH (TFIIH) [17, 18]. Patients with *ERCC3* mutations manifest NER-defective syndromes including xeroderma pigmentosum group B (OMIM 610651) and trichothiodystrophy 2 (OMIM 616390) and have a high increased risk for skin cancer [19, 20].

Moreover, it has been reported that the *ERCC3* gene variants are associated with an elevated risk of developing various types of cancer, including breast cancer, ovarian cancer, lung cancer, osteosarcoma, bladder cancer, colorectal cancer, medulloblastoma, chronic lymphocytic leukemia, malignant pleural mesothelioma, and thyroid cancer (Table 5) [9–12, 21–32, 34]. Multiple multilocus inherited neoplasia alleles syndrome cases with *ERCC3* truncating mutations were reported, which implied that *ERCC3* variants also possibly play a role in modifying the cancer phenotype [11, 31, 35]. *In vitro* study suggested that the recurrent mutation, c.325C > T (p.R109X), can cause reduced *ERCC3* expression and impaired DNA-repair function [9].

In this case-control study among a Han Chinese population composed of 592 participators, we assessed the role of *ERCC3* variants including a novel germline nonsense variant c.348C > G (p.Y116X) and two missense variants rs754010782 (p.R360C) and rs371627165 (p.V471I) in breast cancer pathogenesis. The c.348C > G (p.Y116X) cosegregated with breast cancer in the family. Previous exome sequencing analysis of the proband excluded the *BRCA* mutations, suggesting that this mutation is the most likely genetic cause for this breast cancer family. No frequency bias for the genotype and allele of rs754010782 and rs37162716 was observed in our well-characterized cohort. The truncating mutation p.Y166X is found to potentially increase the risk of breast cancer in mainland Han-Chinese population, while the other two missense variants, rs754010782 and rs371627165, have no apparent association with the development of breast cancer. Some variables, including constraints on the sample size, no matching germline DNA, FFPE-DNA damage, and the infrequency of the genotypes or alleles among the Han-Chinese population, may contribute to the inconclusive outcomes of the two variants rs754010782 and rs371627165. In addition, intrinsic and extrinsic factors, such as epigenetics, geographical origin, and limited sample size, may lower the representativeness of this Han-Chinese population. Further replication studies in a large number of familial breast cancers may provide more genetic evidence for the risk role of p.Y166X mutation.

Up to now, only 13 *ERCC3* truncating and splicing mutations have been reported in a minimum of 29 breast cancer patients and confer a 1.53 ∼ 3.54-fold breast cancer risk [9–12, 24, 30, 31, 33, 36, 37]. Impaired *ERCC3* function in NER may be a potential pathogenesis for breast cancer [9]. In the analysis conducted using bc-GenExMiner v4.9, it was observed that a decrease in *ERCC3* expression is significantly associated with ER, PR, and nontriple-negative status, as well as the nodal status. Studies have also reported a significantly higher frequency of *ERCC3* mutation in ER-positive breast cancer [9, 12]. Confounding factors, including the geographical and ethnic backgrounds of the study population, methodological variations, potential interactions with other gene variants, and environmental factors, as well as additional complicating factors, are overlooked during the analysis. Therefore, they should all be taken into account when interpreting the results of this bioinformatic analysis.

By IHC analysis, *ERCC3* negative expression was identified in the tumor tissues of patients with p.Y116X mutation. The c.348C > G (p.Y116X) mutation may be involved in breast cancer by causing reduction in *ERCC3* expression. This result is consistent with previous studies that *ERCC3* truncating mutation cause decreased *ERCC3* expression [9]. Mild or moderate expression was observed in tumor tissues of patients with rs754010782 and rs371627165 variants. The tumor tissues of patients S-1, S-2, S-3, and S-5 tend to present a slightly decreased *ERCC3* expression. The patient S-4 has the similar *ERCC3* expression level compared with positive external control and normal breast tissues. Influences of rs754010782 and rs371627165 on *ERCC3* expression requires further evidence from more variant carriers.

*ERCC3* truncating mutation has the potential to serve as a biomarker for the pathological diagnosis of breast cancers. By exploring *ERCC3* gene genomic alterations in breast cancers using c-BioPortal, we found that *ERCC3* amplification concentratedly occurred in breast invasive cancer NOS, and most of deep deletions occurred in metaplastic breast cancer. *ERCC3* copy number variant (CNV) detection may help confirm histopathological type of breast cancer and breast cancer metastasis.

## 5. Conclusion

In conclusion, this study revealed that *ERCC3*-truncating mutation p.Y166X may contribute to the breast cancer development in Han-Chinese population. Since the absence of common variant with a high allele frequency in our study, more genetic analysis studies in larger cohorts from different regions as well as functional studies are warranted to estimate the relative risk for breast cancer conferred by *ERCC3* mutations. Our findings also imply that *ERCC3*-truncating mutation and CNV have potential clinical significance in pathological diagnosis of breast cancer.

## Figures and Tables

**Table 1:** Primers for the exons and exon-intron junctions of the *ERCC3* gene.

Exons	Forward primer (5′ ⟶ 3′)	Reverse primer (5′ ⟶ 3′)	Product size (bp)
1	agcggggtcatcttctctct	gtctcccctaggccgagtt	258
2	gggagagatgctggacctg	agctaagggcatgcttacca	416
3	gtggtgttgggcagcttatt	ctccccacaggaaatctgaa	465
4	acccaggcaagaggaagatt	ctgggccacatctcttgttt	409
5	tggtacagatttggcgaagg	tcaagcaatgggtgaagttg	405
6	ccctgctggttctacccttt	gatgccatggctcacagata	416
7	gctttatcccggttgttgac	tccagacacaacagcctgac	403
8	tgtgtgtcgctcagatgtca	ctcagctccagcctgcttac	473
9	gaccagcctgggcaatataa	gcaggtgagcctaagtcctg	410
10	caaactgctggcattacagg	acagccaccttctgcactct	420
11	tgagctcctttttccctctg	caggagaagttccttcagcag	289
12	aaattctggatcagttgacttttt	agaggagtgacctcctgcaa	227
13	cactccagtgcttgcttttg	ggcaaggcctctaatctcct	342
14	gagggaagtgaccaaagcag	ccatccaggcaggactacat	343
15	gactaacatgggctggttcc	gatgcatcttcctcagcaca	300

**Table 2:** Allelic frequencies and *in silico* predictions of the *ERCC3* variants.

Variant		Cases	Samples	Zygosity	dbSNP ID	Allele frequencies				*In silico* predictions				ACMG
Nucleotide change	Amino acid change					ExAC_all	1000 genomes_all	GnomAD_all	ChinaMAP	MutationTaster	PolyPhen-2	Panther	FATHMM	
c.348C > G	p.Y116X	P-1	PBL	Het	—	—	—	—	—	Disease causing	—	Probably damaging	—	LP
c.1078C > T	p.R360C	S-1 and 2	FFPE	Het	rs754010782	3.300 × 10^−5^	—	8.543 × 10^−5^	—	Disease causing	Probably damaging	Probably damaging	Tolerated	US
c.1411G > A	p.V471I	S-3, 4 and 5	FFPE	Het	rs371627165	2.479 × 10^−4^	2.596 × 10^−3^	2.891 × 10^−4^	7.083 × 10^−4^	Disease causing	Benign	Probably damaging	Tolerated	US

PBL, peripheral blood leukocytes; FFPE, formalin fixed and paraffin embedded; dbSNP, the single nucleotide polymorphism database; ExAC_all, exome aggregation consortium; gnomAD_all, genome aggregation database; ChinaMAP, the China metabolic analytics project; PolyPhen-2, polymorphism phenotyping version 2; PANTHER, protein analysis through evolutionary relationships; FATHMM, functional analysis through hidden Markov models; Het, heterozygous; ACMG, the American College of Medical Genetics and Genomics; LP, likely pathogenic; US, uncertain significance.

**Table 3:** Clinicopathological characteristics of patients with *ERCC3* variants.

Variant	Cases	Onset age	Familial history	TNM stage	Tumor location	Grade	IHC		
							ER	PR	HER2
p.Y116X	P-1	31	Y	T2N1M0	Unilateral	III	+	+	−

p.R360C	S-1	35	N	T1N0M0	Unilateral	II	+	−	−
	S-2	38	N	T1N2M0	Unilateral	II	+	−	−

p.V471I	S-3	62	N	T3N1M0	Unilateral	III	+	+	−
	S-4	57	N	T1N0M0	Bilateral	I	+	−	+
	S-5	40	N	T1N0M0	Unilateral	II	+	−	−

IHC, immunohistochemistry; ER, estrogen receptor; PR, progesterone receptor; HER2, human epidermal growth factor 2.

**Table 4:** Genotypic and allelic distributions of *ERCC3* variants in patients and controls.

dbSNP ID	Genotype/allele	Patients	Controls	*P*	OR (95% CI)
—	CC	290	291	—	—
	CG	1	0		
	GG	0	0		
	C	581	582	—	—
	G	1	0		

rs754010782	CC	289	291	0.499	—
	CT	2	0		
	TT	0	0		
	C	580	582	0.500	—
	T	2	0		

rs371627165	GG	288	290	0.624	3.021 (0.312∼29.212)
	GA	3	1		
	AA	0	0		
	G	579	580	1.000	1.503 (0.250∼9.026)
	A	3	2		

OR, odds ratio; CI, confidence interval.

**Table 5:** The cancer-related *ERCC3* gene mutations and variants reported in publications.

Nucleotide change (NM_000122.2)	Amino acid change (NP_000113.1)	Chromosome location (hg38)	dbSNP ID	Related-cancer
c.325C > T [9–11]	p.R109X	chr2: 127292756	rs34295337	Breast cancer
c.335dup [11]	p.H112QfsX4	chr2: 127292746	—	Breast cancer, ovarian cancer
c.471 + 555A > G [21, 22]	—	chr2: 127292055	rs4150407	Bladder cancer, lung cancer
c.583C > T [11]	p.R195X	chr2: 127289763	—	Breast cancer, ovarian cancer
c.658-1G > A [11]	—	chr2: 127289502	—	Breast cancer
c.694A > G [23]	p.T232A	chr2: 127289465	—	Lung cancer
c.700C > T [12, 24]	p.R234X	chr2: 127289396	—	Breast cancer
c.760C > T [11]	p.Q254X	chr2: 127289399	—	Ovarian cancer
c.786_791delAGAAGA [25]	p.E262_E264del	chr2: 27289368_127289373	—	Malignant pleural mesothelioma
c.917T > C [26]	p.I306T	chr2: 127288770	—	Colorectal cancer
c.1300G > T [27]	p.E434X	chr2: 127286745	—	Medulloblastoma
c.1342 + 2179G > A [28]	—	chr2: 127284524	rs4150434	Lung caner
c.1343−2708A > G [29]	—	chr2: 127283339	rs4150441	Osteosarcoma
c.1346A > G [23]	p.K449R	chr2: 127280628	—	Lung cancer
c.1354C > T [12]	p.R452X	chr2: 127280620	—	Breast cancer
c.1421_1422insA [11]	p.D474EfsX2	chr2: 127280553	rs587778281	Breast cancer
c.1720C > T [12]	p.R574X	chr2: 127279183	—	Breast cancer
c.1757delA [10, 30, 31]	p.Q586RfsX25	chr2: 127272935	—	Breast cancer
c.1757_1758delAG [11, 32]	p.Q586RfsX17	chr2: 127272934_127272935	—	Breast cancer, ovarian cancer, thyroid cancer
c.1841C > A [12]	p.S614X	chr2: 127271440	—	Breast cancer
c.1854_1867del [33]	p.E619HfsX24	Chr2: 127271416_127271429	—	Breast cancer
c.2111C > T [34]	p.S704L	chr2: 127259402	rs4150521	Chronic lymphocytic leukemia
c.2218-1G > A [11]	—	chr2: 127257728	—	Breast cancer

## Data Availability

The data used to support the findings of this study are available from the corresponding author upon reasonable request.

## Supplementary Materials

Supplementary Materials Supplementary Figure 1: the hematoxylin-eosin staining of tumor tissues of the affected member. Supplementary Figure 2: the hematoxylin-eosin staining and IHC (ER, PR, and ERCC3) in tumor tissues of the patient P-1, S-1, and S-2 and normal breast tissues. Supplementary Figure 3: the hematoxylin-eosin staining and IHC (ER, PR, and ERCC3) in tumor tissues of the patient S-3, S-4, and S-5 and normal breast tissues.

## References

[1] Sung H., Ferlay J., Siegel R. L. (2021). Global cancer statistics 2020: GLOBOCAN estimates of incidence and mortality worldwide for 36 cancers in 185 countries. *CA: A Cancer Journal for Clinicians*.

[2] Barzaman K., Karami J., Zarei Z. (2020). Breast cancer: biology, biomarkers, and treatments. *International Immunopharmacology*.

[3] Gao Y., Samreen N., Heller S. L. (2022). Non-BRCA early-onset breast cancer in young women. *RadioGraphics*.

[4] Sokolova A., Johnstone K. J., McCart Reed A. E., Simpson P. T., Lakhani S. R. (2023). Hereditary breast cancer: syndromes, tumour pathology and molecular testing. *Histopathology*.

[5] Ellsworth D. L., Turner C. E., Ellsworth R. E. (2019). A review of the hereditary component of triple negative breast cancer: high- and moderate-penetrance breast cancer genes, low-penetrance loci, and the role of nontraditional genetic elements. *Journal of Oncology*.

[6] Liao H., Zhang J., Zheng T. (2022). Identification of mutation patterns and circulating tumour DNA-derived prognostic markers in advanced breast cancer patients. *Journal of Translational Medicine*.

[7] Michailidou K., Lindström S., Dennis J. (2017). Association analysis identifies 65 new breast cancer risk loci. *Nature*.

[8] Wendt C., Margolin S. (2019). Identifying breast cancer susceptibility genes-a review of the genetic background in familial breast cancer. *Acta Oncologica*.

[9] Vijai J., Topka S., Villano D. (2016). A recurrent ERCC3 truncating mutation confers moderate risk for breast cancer. *Cancer Discovery*.

[10] Maxwell K. N., Hart S. N., Vijai J. (2016). Evaluation of ACMG-guideline-based variant classification of cancer susceptibility and non-cancer-associated genes in families affected by breast cancer. *The American Journal of Human Genetics*.

[11] Stradella A., Del Valle J., Rofes P. (2020). ERCC3, a new ovarian cancer susceptibility gene?. *European Journal of Cancer*.

[12] Palmer J. R., Polley E. C., Hu C. (2020). Contribution of germline predisposition gene mutations to breast cancer risk in African American women. *Journal of the National Cancer Institute: Journal of the National Cancer Institute*.

[13] Shiovitz S., Korde L. A. (2015). Genetics of breast cancer: a topic in evolution. *Annals of Oncology*.

[14] Tan P. H., Ellis I., Allison K. (2020). The 2019 World Health Organization classification of tumours of the breast. *Histopathology*.

[15] Criscitiello C., Corti C. (2022). Breast cancer genetics: diagnostics and treatment. *Genes*.

[16] Torabi Dalivandan S., Plummer J., Gayther S. A. (2021). Risks and function of breast cancer susceptibility alleles. *Cancers*.

[17] Fan L., DuPrez K. T. (2015). XPB: an unconventional SF2 DNA helicase. *Progress in Biophysics and Molecular Biology*.

[18] Fuss J. O., Tainer J. A. (2011). XPB and XPD helicases in TFIIH orchestrate DNA duplex opening and damage verification to coordinate repair with transcription and cell cycle via CAK kinase. *DNA Repair*.

[19] Hu Z., Xu L., Shao M. (2006). Polymorphisms in the two helicases ERCC2/XPD and ERCC3/XPB of the transcription factor IIH complex and risk of lung cancer: a case-control analysis in a Chinese population. *Cancer Epidemiology, Biomarkers & Prevention*.

[20] Shah P., He Y. Y. (2015). Molecular regulation of UV-induced DNA repair. *Photochemistry and Photobiology*.

[21] Feki-Tounsi M., Khlifi R., Louati I. (2017). Polymorphisms in XRCC1, ERCC2, and ERCC3 DNA repair genes, CYP1A1 xenobiotic metabolism gene, and tobacco are associated with bladder cancer susceptibility in Tunisian population. *Environmental Science and Pollution Research*.

[22] Buch S. C., Diergaarde B., Nukui T. (2012). Genetic variability in DNA repair and cell cycle control pathway genes and risk of smoking-related lung cancer. *Molecular Carcinogenesis*.

[23] Matakidou A., Eisen T., Fleischmann C., Bridle H., Houlston R. S. (2006). Evaluation of xeroderma pigmentosum XPA, XPC, XPD, XPF, XPB, XPG and DDB2 genes in familial early-onset lung cancer predisposition. *International Journal of Cancer*.

[24] Jalkh N., Chouery E., Haidar Z. (2017). Next-generation sequencing in familial breast cancer patients from Lebanon. *BMC Medical Genomics*.

[25] Betti M., Casalone E., Ferrante D. (2017). Germline mutations in DNA repair genes predispose asbestos-exposed patients to malignant pleural mesothelioma. *Cancer Letters*.

[26] de Voer R. M., Hahn M. M., Weren R. D. (2016). Identification of novel candidate genes for early-onset colorectal cancer susceptibility. *PLoS Genetics*.

[27] Waszak S. M., Northcott P. A., Buchhalter I. (2018). Spectrum and prevalence of genetic predisposition in medulloblastoma: a retrospective genetic study and prospective validation in a clinical trial cohort. *The Lancet Oncology*.

[28] Huang Y., Meng C., Long W. (2019). [XP gene polymorphisms and haplotypes with genetic susceptibility to lung cancer]. *Wei Sheng Yan Jiu*.

[29] Xu Q., Zhang Z., Sun W., Hu B. (2017). Haplotype analysis on relationship of ERCC2 and ERCC3 gene polymorphisms with osteosarcoma risk in Chinese young population. *Mammalian Genome*.

[30] Tedaldi G., Tebaldi M., Zampiga V. (2017). Multiple-gene panel analysis in a case series of 255 women with hereditary breast and ovarian cancer. *Oncotarget*.

[31] Bonache S., Esteban I., Moles-Fernández A. (2018). Multigene panel testing beyond BRCA1/2 in breast/ovarian cancer Spanish families and clinical actionability of findings. *Journal of Cancer Research and Clinical Oncology*.

[32] Guan Y., Hu H., Peng Y. (2015). Detection of inherited mutations for hereditary cancer using target enrichment and next generation sequencing. *Familial Cancer*.

[33] Su Y., Yao Q., Xu Y. (2021). Characteristics of germline non-BRCA mutation status of high-risk breast cancer patients in China and correlation with high-risk factors and multigene testing suggestions. *Frontiers in Genetics*.

[34] Rudd M. F., Sellick G. S., Webb E. L., Catovsky D., Houlston R. S. (2006). Variants in the ATM-BRCA2-CHEK2 axis predispose to chronic lymphocytic leukemia. *Blood*.

[35] Dumbrava E. I., Brusco L., Daniels M. (2019). Expanded analysis of secondary germline findings from matched tumor/normal sequencing identifies additional clinically significant mutations. *JCO Precision Oncology*.

[36] Einhorn Y., Weissglas-Volkov D., Carmi S., Ostrer H., Friedman E., Shomron N. (2017). Differential analysis of mutations in the Jewish population and their implications for diseases. *Genetics Research*.

[37] Foley S. B., Rios J. J., Mgbemena V. E. (2015). Use of whole genome sequencing for diagnosis and discovery in the cancer genetics clinic. *EBioMedicine*.

