# Supplementary material for: ERCC3 Gene Associated with Breast Cancer: A Genetic and Bioinformatic Study
Source: Breast J. 2024 Jul 14;2024:7278636. doi: 10.1155/2024/7278636 (PMC11260512; doi:10.1155/2024/7278636)

**Supplementary Figure 1: The hematoxylin-eosin staining of tumor tissues of the affected member. The specimen of the affected member was diagnosed to nonspecific invasive carcinoma (grade Ⅱ) in the right breast.**

**Supplementary Figure 2: The hematoxylin-eosin staining and IHC (ER, PR and ERCC3) in tumor tissues of the patient P-1, S-1 and S-2, and normal breast tissues. (a) The hematoxylin-eosin staining in tumor tissues of the P-1. (b) ER expression in tumor tissues of the P-1 by IHC. (c) PR expression in tumor tissues of the P-1 by IHC. (d) ERCC3 expression in tumor tissues of the P-1 by IHC. (e) The hematoxylin-eosin staining in tumor tissues of the S-1. (f) ER expression in tumor tissues of the S-1 by IHC. (g) PR expression in tumor tissues of the S-1 by IHC. (h) ERCC3 expression in tumor tissues of the S-1 by IHC. (i) The hematoxylin-eosin staining in tumor tissues of the S-2. (j) ER expression in tumor tissues of the S-2 by IHC. (k) PR expression in tumor tissues of the S-2 by IHC. (l) ERCC3 expression in tumor tissues of the S-2 by IHC. (m) The hematoxylin-eosin staining in normal breast tissues. (n) ER expression in normal breast tissues by IHC. (o) PR expression in** **normal breast tissues by IHC. (p) Wild-type ERCC3 expression in normal breast tissues by IHC.**

**
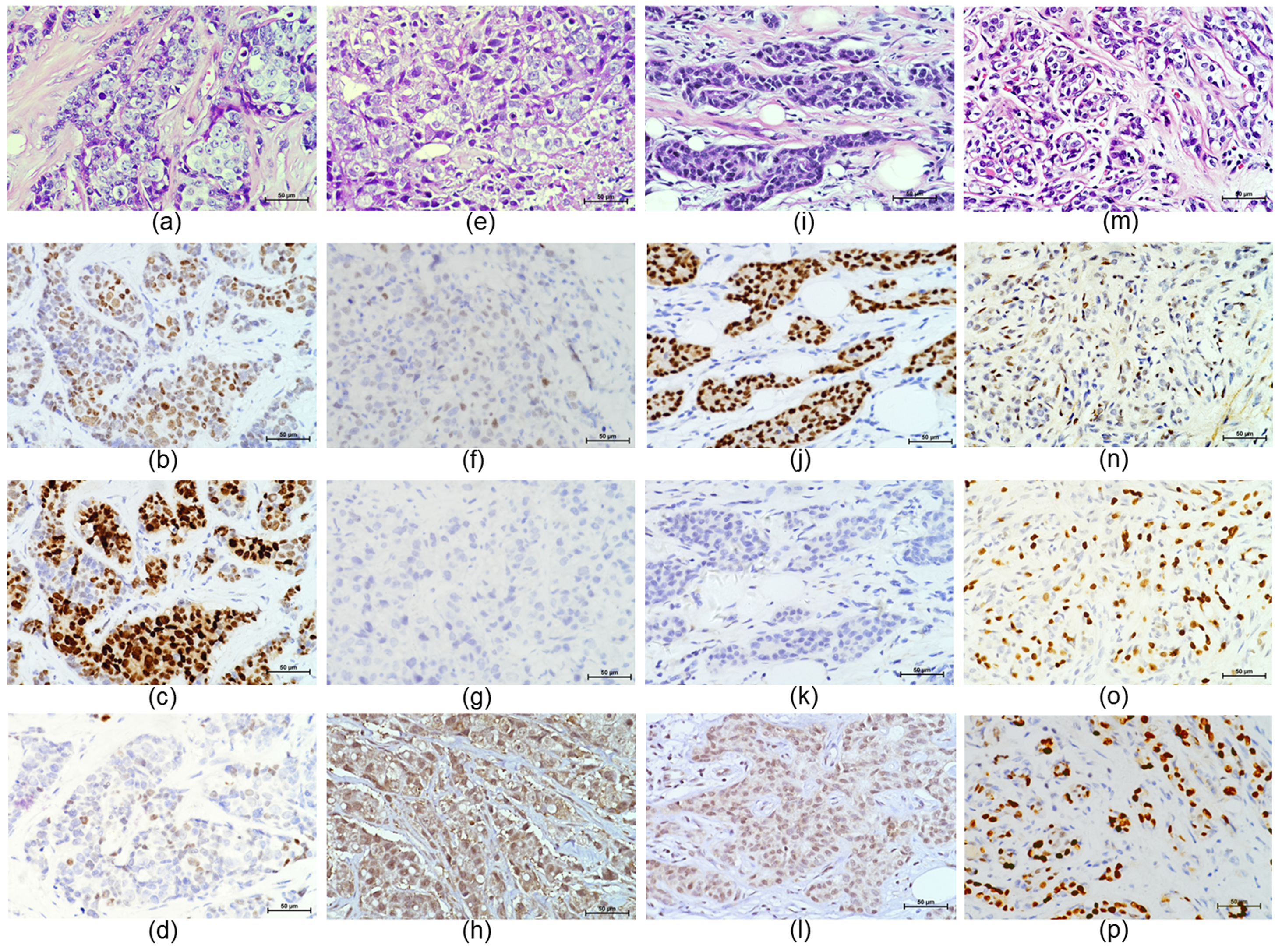
**

**Supplementary Figure 3: The hematoxylin-eosin staining and IHC (ER, PR and ERCC3) in tumor tissues of the patient S-3, S-4 and S-5, and normal breast tissues. (a) The hematoxylin-eosin staining in tumor tissues of the S-3. (b) ER expression in tumor tissues of the S-3 by IHC. (c) PR expression in tumor tissues of the S-3 by IHC. (d) ERCC3 expression in tumor tissues of the S-3 by IHC. (e) The hematoxylin-eosin staining in tumor tissues of the S-4. (f) ER expression in tumor tissues of the S-4 by IHC. (g) PR expression in tumor tissues of the S-4 by IHC. (h) ERCC3 expression in tumor tissues of the S-4 by IHC. (i) The hematoxylin-eosin staining in tumor tissues of the S-5. (j) ER expression in tumor tissues of the S-5 by IHC. (k) PR expression in tumor tissues of the S-5 by IHC. (l) ERCC3 expression in tumor tissues of the S-5 by IHC. (m) The hematoxylin-eosin staining in normal breast tissues. (n) ER expression in normal breast tissues by IHC. (o) PR expression in** **normal breast tissues by IHC. (p) Wild-type ERCC3 expression in normal breast tissues by IHC.**


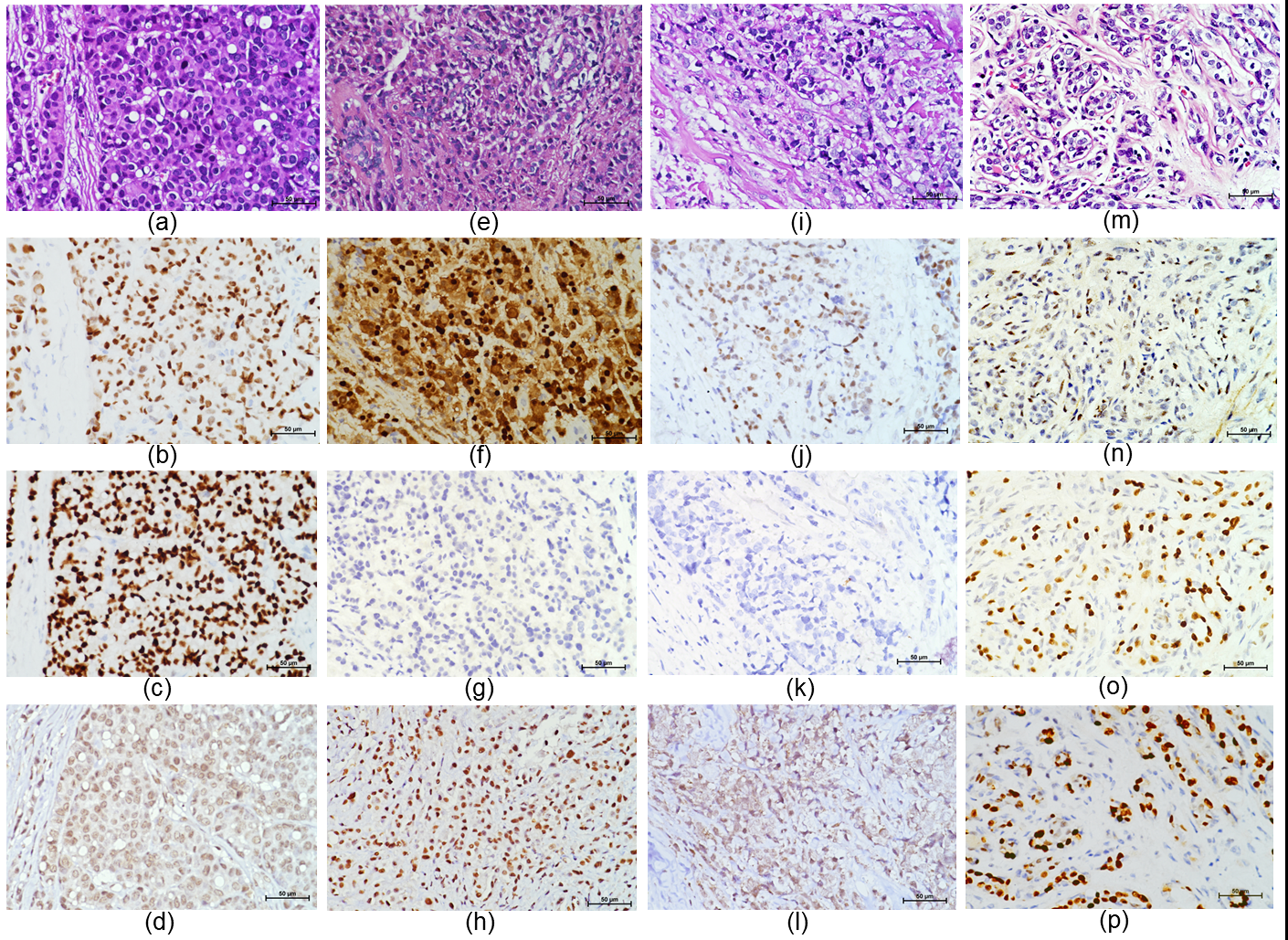

Supplement: Supplementary Materials — Supplementary Figure 1: the hematoxylin-eosin staining of tumor tissues of the affected member. Supplementary Figure 2: the hematoxylin-eosin staining and IHC (ER, PR, and ERCC3) in tumor tissues of the patient P-1, S-1, and S-2 and normal breast tissues. Supplementary Figure 3: the hematoxylin-eosin staining and IHC (ER, PR, and ERCC3) in tumor tissues of the patient S-3, S-4, and S-5 and normal breast tissues. [file 7278636.f1.doc]
